# Epidemiology and Long-Term Survival in HIV-Infected Patients With *Pneumocystis jirovecii* Pneumonia in the HAART Era: Experience in a University Hospital and Review of the Literature

**DOI:** 10.1097/MD.0000000000000681

**Published:** 2015-03-27

**Authors:** Cristina López-Sánchez, Vicenç Falcó, Joaquin Burgos, Jordi Navarro, María Teresa Martín, Adrià Curran, Lucía Miguel, Inma Ocaña, Esteve Ribera, Manel Crespo, Benito Almirante

**Affiliations:** From the Department of Infectious Diseases (CL-S, VF, JB, JN, AC, LM, IO, ER, MC, BA); and Department of Microbiology (MTM), University Hospital Valld’Hebron, UniversitatAutònoma de Barcelona, Barcelona, Spain.

## Abstract

As highly active antiretroviral treatment (HAART) is widely available, the incidence of *Pneumocystis jirovecii* pneumonia (PJP) has decreased significantly but still represents a significant cause of morbidity and mortality in developed countries.

We analyzed all the cases with PJP in human immunodeficiency virus (HIV)-infected patients from 2000 to 2013 in a university hospital in Barcelona, Spain, and conducted a systematic literature review to evaluate data regarding incidence, mortality, and long-term survival after PJP in developed settings.

One hundred thirty-six episodes of PJP were analyzed. During the study period, the incidence decreased significantly (from 13.4 cases/1000 patients-year to 3.3 cases/1000 patients-year, *P* < 0.001). Oppositely, median age of the patients increased from 34 years in 2000 to 45 in 2013 (*P *= 0.024). PJP preceded HIV diagnosis in nearly 50% of the cases. Fifteen (11%) patients died during the PJP episode. The main risk factor for in-hospital mortality in our cohort was age >50 years (odds ratio 4.96, 95% confidence interval [CI] 1.45–15.14). Patients who survived were followed-up during a mean time of 44 months. Overall 5-year survival of patients after hospital discharge was 73%. Survival likelihood was 54% higher (88% [95% CI 81–96]) among HAART-adherent patients.

Mean age and the proportion of patients with unknown HIV infection at the time of PJP diagnosis have increased in developed countries in the HAART era. Although the incidence has decreased, in-hospital mortality remains stable in this setting. Long-term survival is very high among HAART-adherent patients.

## 1 INTRODUCTION

The incidence of *Pneumocystis jirovecii* pneumonia (PJP) in developed countries has experienced a significant and progressive decline since highly active antiretroviral treatment (HAART) became available. Soon in the early HAART era, a national surveillance study including 8 cities in the United States demonstrated a decrease in the incidence of PJP from 13 cases per 100 patients-year in 1994 to 3 cases per 100 patients-year in 1997 in human immunodeficiency virus (HIV)-infected patients.^1^ Similarly, PJP incidence decreased by 30% in the Swiss Cohort Study from 1995 to 1997.^2^ However, and despite the effectiveness of primary chemoprophylaxis and the improvements in HAART, opportunistic infections (OIs) continue to be present.^3^ In fact, PJP still represents the most common acquired immunodeficiency syndrome (AIDS)-defining OI in the United States and was reported as the second AIDS-related cause of mortality in a large French cohort.^4–6^ Most of these cases in industrialized countries occur either in people who are unaware of their HIV infection or in patients with poor adherence to HAART.^3,7^

Another important issue is that, despite the improvement of intensive care support, hospital-related mortality of patients diagnosed with PJP remains stable around 10%.^8,9^ On the other hand, data on long-term survival following a PJP episode are scarce.

In this study, we aimed to describe the actual epidemiological trends of patients with HIV infection who develop PJP and evaluate in-hospital mortality and long-term survival. Additionally, we carried out a systematic review of the literature in order to assess the information regarding PJP published in the HAART era in the setting of developed countries.

## 2 PATIENTS, MATERIAL, AND METHODS

### 2.1 Study Design

This is a descriptive cohort study of all HIV-infected adult patients consecutively diagnosed with PJP between January 2000 and December 2013 at the University Hospital Valld’Hebron, in Barcelona, Spain. Our institution is a 1000-bed tertiary hospital where approximately 2100 patients with HIV infection are regularly followed.

### 2.2 Study Variables and Data Collection

Demographic, clinical, laboratory, radiological and microbiological data, antiretroviral therapy, clinical course, and mortality were recorded from each episode. Patients were followed up to 5 years, until death or lost to follow-up or until December 31 of 2013, when the database was closed. All clinical data of HIV-infected patients are routinely included in a database as a part of a continuous observational study. Data analyzed in the present study have been taken from this database. This study was approved by the Ethics Committee of Valld’Hebron Research Institute.

### 2.3 Definitions

A definite diagnosis of PJP was done in the presence of suggestive clinical and radiological findings and demonstration of *Pneumocystis jirovecii* (PJ), either by positive antigen or cysts visualization in bronchoalveolar lavage (BAL) fluid-staining technique.

In those patients in whom fibrobronchoscopy (FBS) could not be performed, a probable diagnosis of PJP was assumed if all the following conditions were fulfilled: CD4 lymphocyte count <200 cells/μL; clinical and radiological findings suggestive of PJP; no prophylaxis against PJP; good response to specific PJP treatment; and exclusion of other pulmonary pathogens, mainly bacterial pneumonia and tuberculosis.

Respiratory failure was defined as oxygen saturation <90% using a pulse oximeter or partial pressure of oxygen <60 mm Hg in arterial blood.

### 2.4 Statistical Analysis

Continuous variables are expressed as the median and interquartile range or mean and standard deviation and were compared using Student *t* test or Mann–Whitney U test as appropriate. Categorical variables were compared using a χ^2^ test or Fisher exact test. The annual incidence of PJP was calculated using the number of HIV-infected patients alive registered yearly in the database from our hospital as denominator, and is expressed as cases per 1000 HIV-infected patients per year. Changes in PJP annual incidence and in the proportion of patients with unknown HIV infection were calculated using the Mantel–Haenszel trend test. Annual changes in patients’ median age were calculated using Kruskal–Wallis test.

A stepwise forward logistic regression model analysis was performed to identify independent risk factors within those variables related to hospital mortality. We excluded from the regression analysis variables with high colinearity by Spearman test (*P* < 0.001 for bivariate correlation). We have also performed a multivariable analysis in order to identify variables associated to bad adherence to HAART during follow-up.

For long-term mortality analysis, patients who died and those lost to follow-up were considered together. Survival distribution was estimated using the Kaplan–Meier method with log-rank test for group comparison. In the follow-up study, we considered a patient to be nonadherent to HAART if he missed ≥2 treatment appointments.

Statistical analyses were performed using the statistical software package IBM SPSS Statistics for Macintosh, Version 21.0 (IBM Corp., Armonk, NY).

### 2.5 Systematic Literature Review: Search Strategy

For this systematic review, we followed the preferred reporting items for systematic reviews and meta-analysis guidelines.^10^ A search in the PubMed database was done using the search string “(*Pneumocystis carinii* pneumonia or *Pneumocystis jirovecii* pneumonia) and (HIV or AIDS).”

We restricted our review to studies published from January 1, 2000, when HAART was widely used in developed countries, to June 1, 2014. Results were limited to human studies, adult patients (≥ 18 years old) and the English language. By abstract review, we selected those articles that included information regarding at least 1 of the following items: incidence rates, patients’ age, proportion of patients with previously undiagnosed HIV infection, mortality, and/or long-term survival. Case reports and reviews were excluded from further analysis.

In studies that comprised patients from the pre-HAART and post-HAART era, only data from patients with PJP diagnosed during the latter were considered for analysis. Because significant epidemiological and clinical differences among PJP between industrialized and developing countries were expected to be found, studies carried out in limited resource settings were not considered for this analysis. Studies that included exclusively non-HIV patients were also excluded. Studies with both HIV and non-HIV-infected population were included only if data of HIV-infected patients could be analyzed separately. Studies were required to have at least 20 PJP cases. Moreover, those studies that included exclusively patients admitted to intensive care units (ICU) were also excluded to avoid a severity bias.

## 3 RESULTS

### 3.1 Baseline Characteristics

One hundred thirty-six HIV-infected patients were diagnosed with PJP between January 2000 and December 2013. There were 94 (69.1%) males and 42 (30.9%) women with a mean age of 41 (±10) years. Fifty percent of them had not been previously diagnosed with HIV at the time of PJP diagnosis. On the other hand, most of the patients who had previously known HIV infection did not follow medical controls or ART. All the demographic and immunologic characteristics of the patients at baseline are summarized in Table 1.

**TABLE 1:**
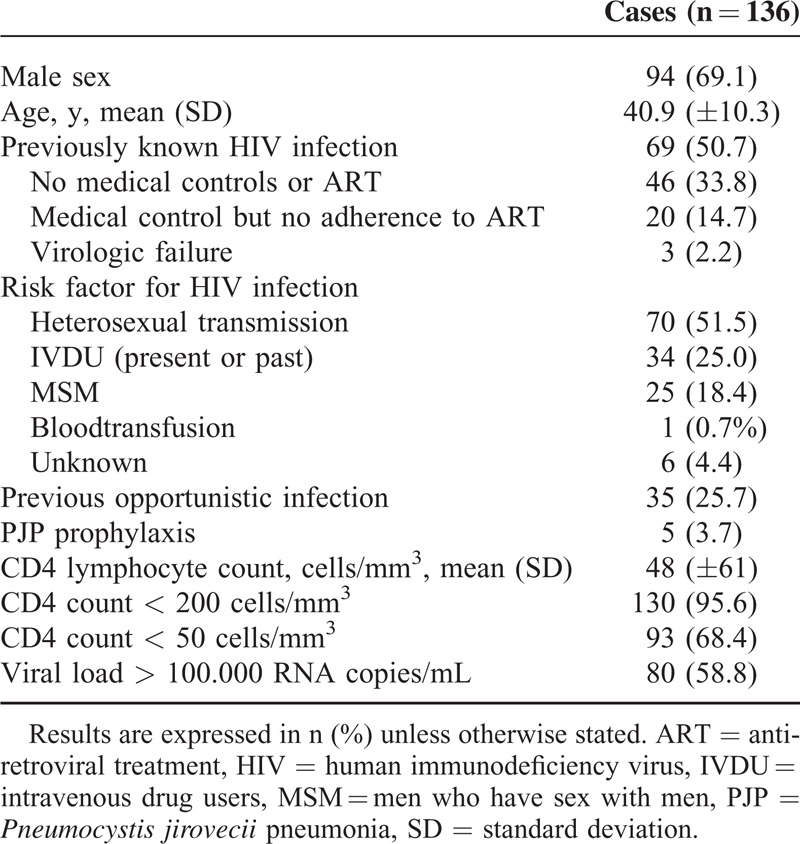
Demographic Characteristics and Immunological Status of All Patients With PJP

### 3.2 Epidemiological Data

**FIGURE 1:**
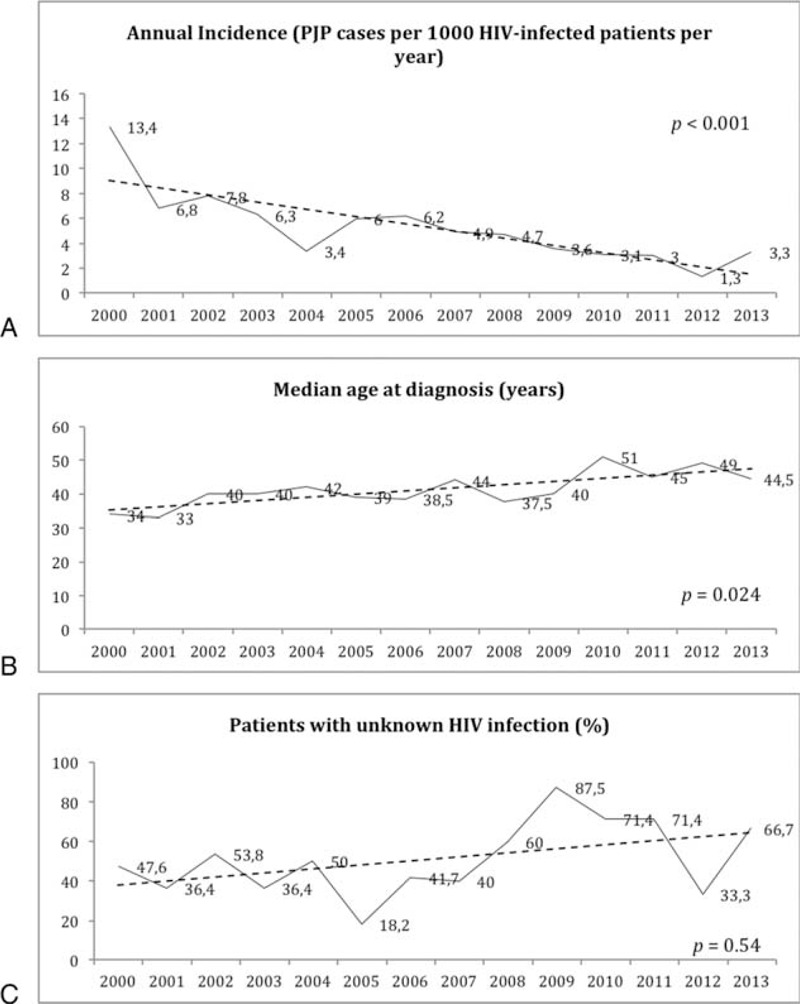
(A) Yearly evolution of annual incidence, (B) median age of the patients at diagnosis, and (C) proportion of patients with unknown HIV infection prior to the PJP episode. HIV = human immunodeficiency virus, PJP = *Pneumocystis jirovecii* pneumonia.

Annual incidence of PJP cases is shown in Figure 1A. We observed a significant decline from 13.4 cases per 1000 HIV-infected patients per year in 2000 to 3.3 cases per 1000 HIV-infected patients per year in 2013 (*P* < 0.001). Median age at diagnosis increased from 34 (30–39) years in 2000 to 44 (35–58) years in 2013 (*P* = 0.024) (Figure 1B). Although not statistically significant, the proportion of patients with unknown HIV-infection increased from 48% on 2000 to 67% in 2013 (*P* = 0.54) (Figure 1C). PJP preceded HIV diagnosis more frequently in patients >50 years old (87% vs 62%, *P* < 0.001).

### 3.3 Clinical Data and Diagnosis

Clinical and diagnosis-related characteristics of PJP episodes are shown in Table 2. The mean average time of symptoms before the patient attended in to the hospital was 22 (±23) days. One hundred eight (79%) patients underwent FBS. Ninety-eight (72%) episodes were considered definite diagnoses of PJP and 38 (28%) were considered probable diagnoses according to the definitions stated earlier. No differences between patients with definite and probable diagnosis regarding to age, previously known HIV infection, immunologic situation at diagnosis, presence of respiratory failure, and outcome were observed (data not shown).

**TABLE 2:**
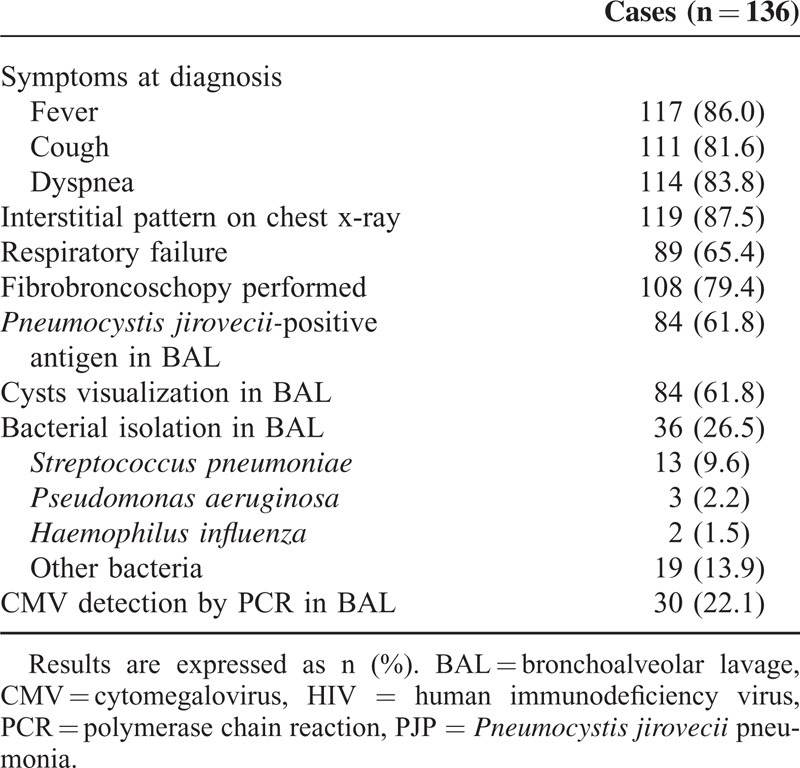
Clinical and Diagnosis-Related Characteristics of All PJP Episodes

### 3.4 In-Hospital Mortality

All patients except for 2 cases treated with intravenous pentamidine, received intravenous trimetroprim–sulfametoxazol as first-choice treatment, and 106 (78%) received corticoid adjunctive treatment. Twenty-eight (21%) patients were admitted to the ICU. Fourteen (10%) of them required invasive mechanical ventilation. Patients >50 years old required ICU admission more often than younger patients (39% vs 27%, *P* = 0.016) and presented more frequently, respiratory failure (82% vs 62%, *P* = 0.058).

The overall related mortality was 11% (15 out of 136 patients). Age >50 years (*P* = 0.004), respiratory failure (*P* = 0.019), ICU admission (*P* < 0.001), and mechanical ventilation (*P* < 0.001) were significantly associated with in-hospital mortality in the unvaried analysis. By multivariate analysis, a trend to higher mortality was observed among patients with respiratory failure. However, age >50 years remained as the only variable independently associated with in-hospital mortality (odds ratio [OR] 4.96, 95% CI 1.45–15.14). In this model, ICU admission and mechanical ventilation were not included because of the high colinearity observed with respiratory failure (*P* < 0.001 for bivariate correlation) (Table 3).

**TABLE 3:**
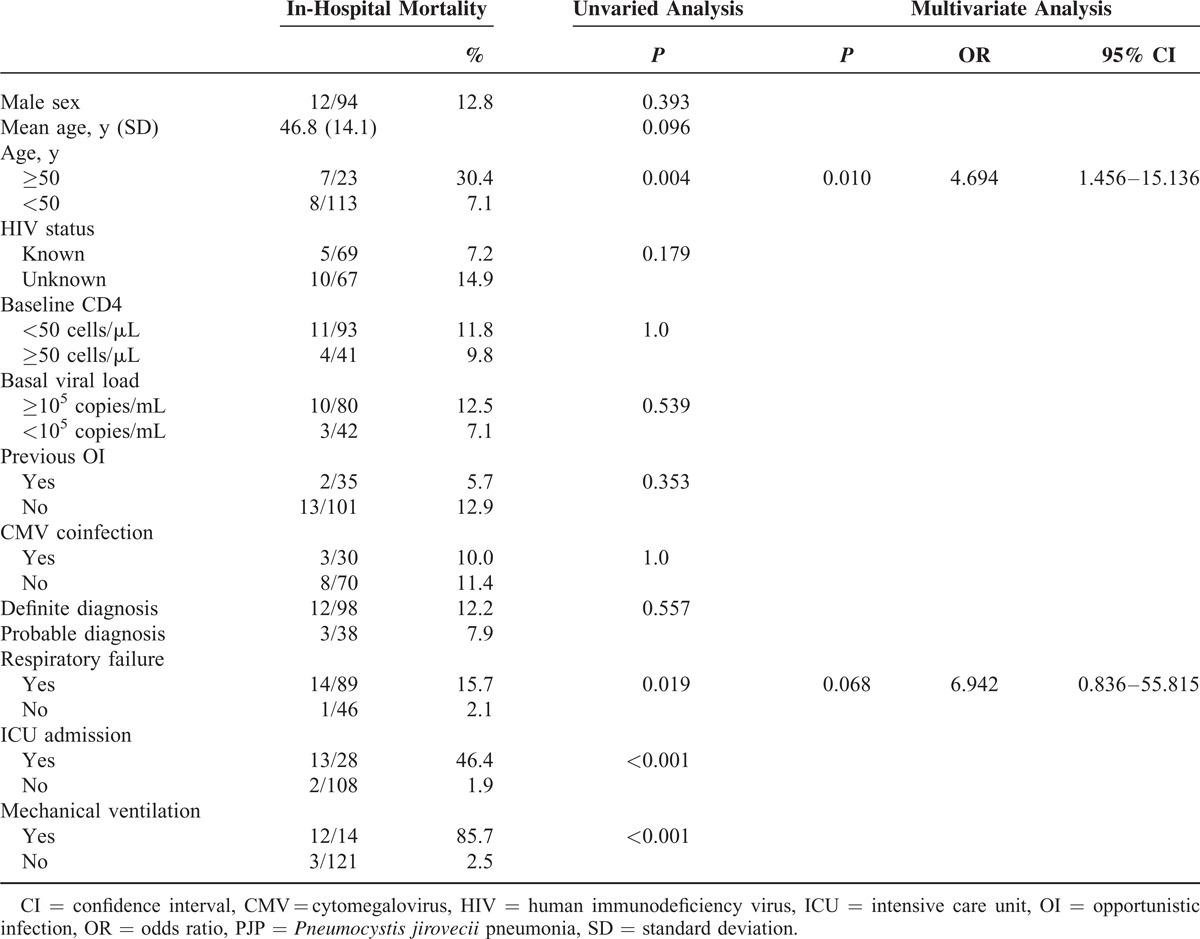
Risk-Factor Analysis for In-Hospital Mortality

### 3.5 Follow-Up

**FIGURE 2:**
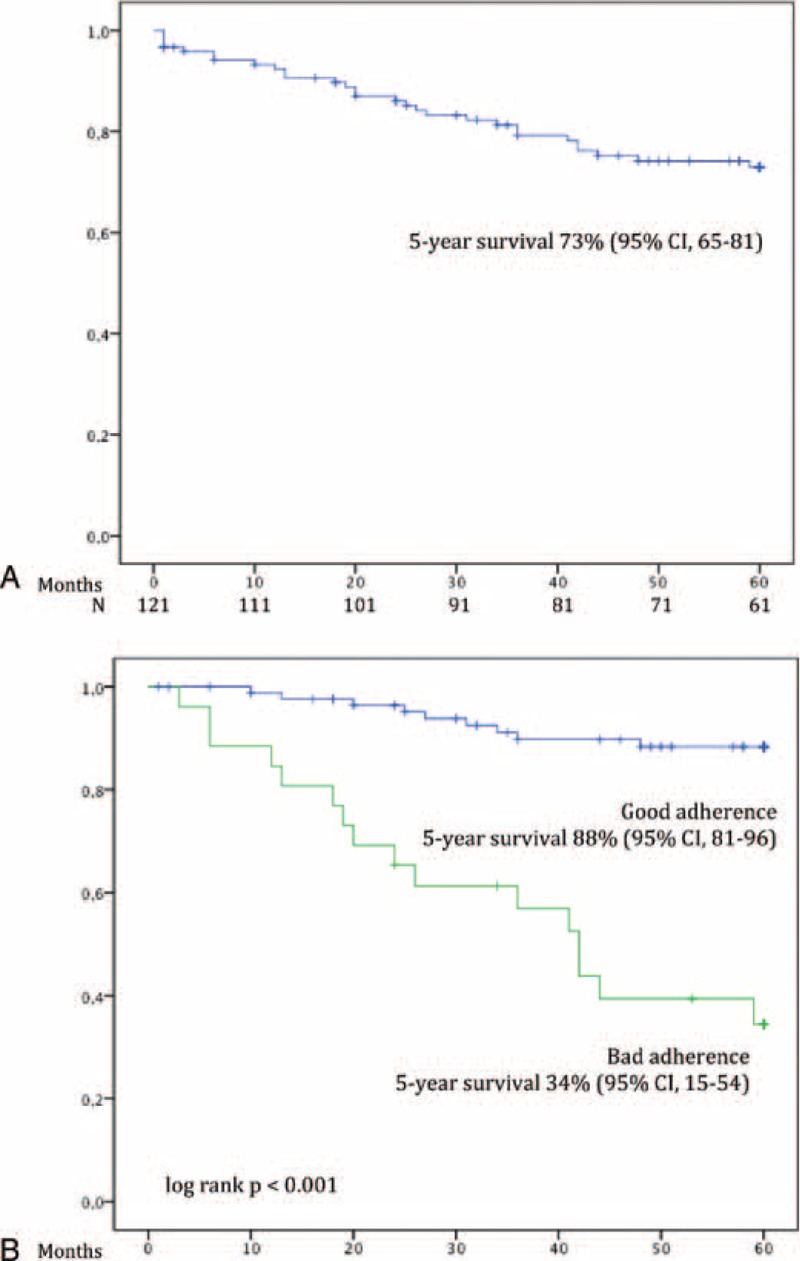
(A) Overall long-term survival of patients with PJP after hospital discharge and (B) long-term survival of patients with PJP after hospital discharge stratified by adherence to HAART. HAART = highly active antiretroviral treatment, PJP = *Pneumocystis jirovecii* pneumonia.

After a mean follow-up of 44 months after hospital discharge, 68% (92 out of 121 patients) survived. HAART initiation was offered to all of them, although only 111 (91.7%) of them accepted treatment initiation. Twenty (16%) patients died and 9 (7%) were lost to follow-up. Death was attributable to AIDS-related complications in 14 cases. The 5-year survival probability was 73% (95% CI 64.6–81.4) (Figure 2A). According to the definitions stated above, at 5-year follow-up, 91 (75.2%) patients presented good adherence to HAART and 30 (24.8%) patients presented bad adherence to HAART. Poor adherence to HAART was significantly associated with lower 5-year survival (Figure 2B). In the multivariate analysis, the only factor associated with bad adherence to HAART was intravenous drug use (IVDU) as risk factor for HIV infection (OR 5.87, 95% CI 2.2–15.6).

### 3.6 Systematic Literature Review

**FIGURE 3:**
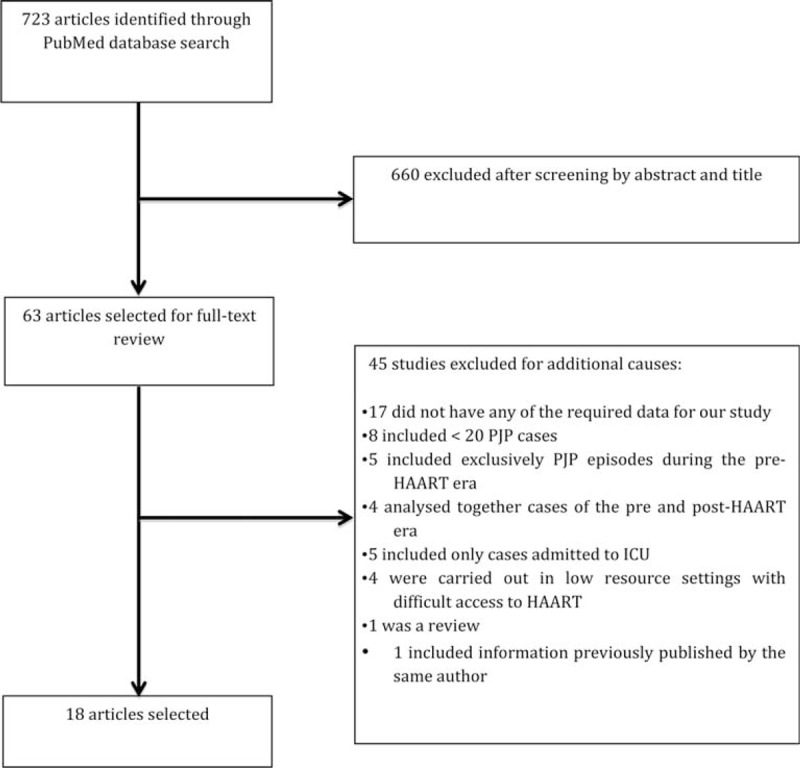
Flow chart of search strategy for article selection. ICU = intensive care unit.

Initially, 723 articles were identified and screened through PubMed database. By title and abstract review, 660 articles were excluded on the grounds of being single-case reports, reviews, or guidelines, or having a focus other than HIV-infected patients. From the remaining 63 studies, 45 were excluded for additional causes after full-text reading (Figure 3). The key studies addressing PJP in the developed world are summarized in Table 4.

**TABLE 4:**
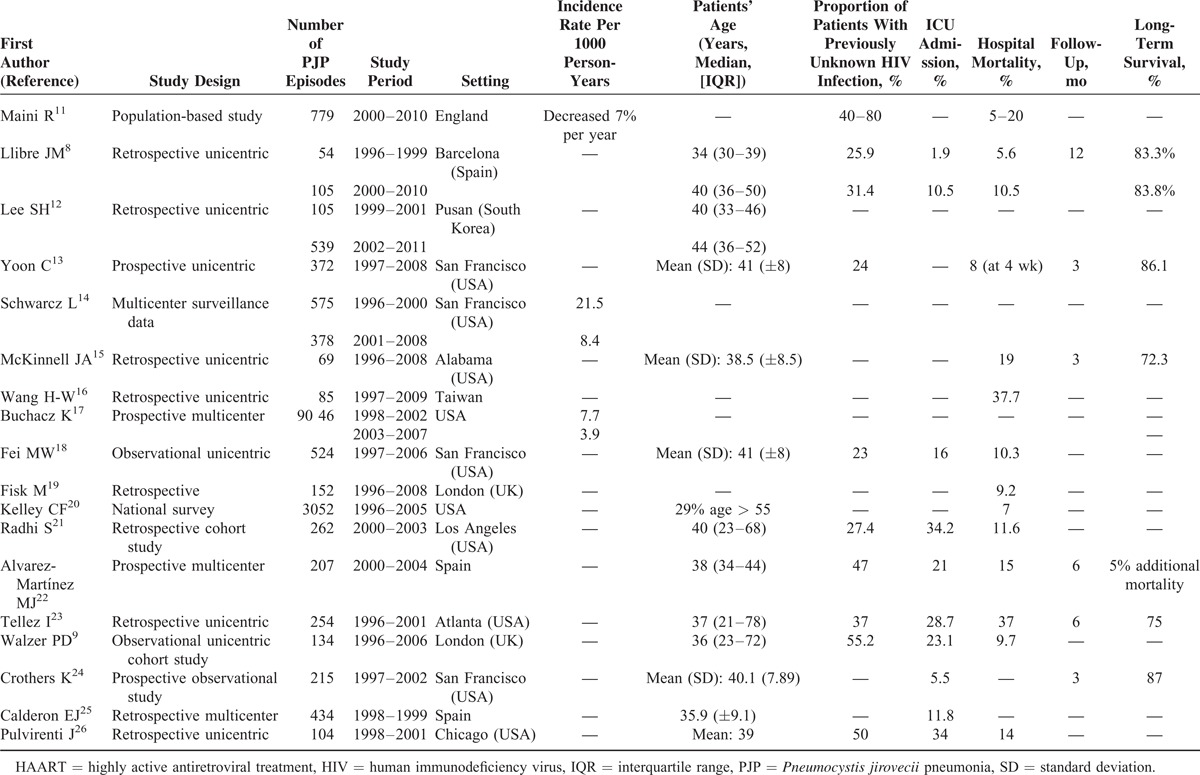
Summary of Selected Studies on PJP in HIV-Infected Patients in Developed Settings During the HAART Era

Only 3 studies included information about the evolution of the incidence of PJP and in all 3, a negative trend can be observed throughout the years. A study based on patients from the National Health Service in England reported a decrease in the incidence of 7% each year.^11^ Similarly, 2 large multicenter north-American studies described higher incidence rates per 1000 person-years during the early HAART period than in the following years.^14,17^ Regarding age, 2 studies compared the median age between the early and late-HAART era and both reported an increase of the median age of patients throughout the years.^8,12^ The proportion of patients with unknown HIV infection prior to the PJP episode varies widely among the different studies ranging from 23% to 80%.^8,9,11,13,21–23,26^

Most of the selected studies report in-hospital mortality rates between 10% and 20%, with an apparent stability from 2000 onwards.^8,9,11,13,15,18,20–22,26^ Higher in-hospital mortality rates, up to 37%, were reported from 2 studies.^16,23^

Several factors have been identified as risk factors for in-hospital mortality. The leading factor associated with higher in-hospital mortality is respiratory failure with adjusted OR that ranges from 2.34 to 6.47.^8,9,16,18,26^ The need for mechanical ventilation or ICU admission, as surrogate markers of respiratory failure, have also been associated with death in other studies.^8,21,24^ Albumin levels <3 g/dL follow respiratory failure as the second most important risk factor for in-hospital mortality with an increased risk of mortality between 3.63 to 4.62 fold.^18,21,24^ Finally, age was found to be associated with an increased risk of mortality, by 1.54 and 1.69 per every 10 years increase, in 2 studies.^9,18^ Other less commonly identified risk factors for mortality are low systolic blood pressure, lymphocyte count ≤10%, recent injection drug use, high bilirubin levels, pneumothorax, presence of medical comorbidity, and concomitant pulmonary Kaposi sarcoma.^9,16,18,21^

There are scarce published data regarding long-term survival of HIV-infected patients after PJP infection. We have only identified 5 studies with short follow-up periods, between 3 and 6 months, reporting survival percentages from 72.3% to 87%.^13,15,22–24^ Another study reported an 83.3% survival rate at 12 months of follow-up.^8^

## 4 DISCUSSION

In the last years, and since HAART is available in developed countries, the concerns of physicians attending HIV-infected patients have evolved from AIDS-specific complications to the so-called “non-AIDS events.” Nevertheless, and despite a clear decrease in their incidence, opportunistic infections, including PJP, still represent an important cause of morbidity and mortality in HIV-infected patients, not only in developing countries but also in developed settings.^4–6,19^

Our study reflects, in the same way as other studies, a significant reduction in the annual incidence of cases of PJP from 13.4 cases per 1000 HIV-infected patient/years in 2000 to 3.3 cases in 2013.^1,2^ Furthermore, 2 epidemiological trends deserve attention. On the one hand, the proportion of patients in which the PJP episode preceded HIV diagnosis has substantially increased from 47.6% in 2000 to 66.7% in 2013 according to our results. These data are a reflection of the problem of patients who are unaware of their HIV infection and present late in the course of the disease. As late HIV diagnosis is associated with an increased risk of morbidity and mortality, as well as increased rates of HIV transmission, encouraging strategies of early diagnosis are mandatory.

On the contrary, the age of patients presenting with PJP is also increasing. In accordance with our results, a national north-American study, demonstrated an increasing proportion of the patients >55 years old, from 21% during the period 1986–1989 to 29% in 1996–2005.^20^ In the same way, in the series reported by Walzer *et al*,^9^ 28% of the patients were ≥50 years old.^9^ These data are remarkable as older age at presentation implies a poor prognosis. In keeping with previous data,^9,18^ in our study, older age was the only predictor of PJP outcome, and risk of mortality was 5 times higher in patients >50 years old as compared to younger patients. Other studies have also documented higher risk of mortality among patients aged ≥50 years. Although in these studies, age was not an independent predictor of mortality after adjustment for severity of PJP infection, the authors alerted about the fact that higher mortality risk in older patients with PJP might be related to a more severe presenting illness, underrecognition of HIV infection, and delay in starting PJP-specific therapy.^27–29^ Indeed, in our study, the percentage of patients with unknown HIV infection was higher among those ≥50 years (87%) compared with younger patients (42%, *P* < 0.001) and older patients needed ICU more frequently (39% vs 17%, *P* = 0.016).

The overall in-hospital mortality in our study was 11%, similar to that reported in other series in the HAART era.^8,9,18,21^ Aside from older age at presentation, other leading risk factors for in-hospital mortality described in literature are respiratory failure and hypoalbuminemia.

In our cohort, patients who survived to the episode were followed-up during a mean time period of 44 months. Overall, the probability of survival after hospital discharge was 94% and73% at 1 and 5 years, respectively. In the early HAART era, Dworkin *et al*^30^ observed that a poor immunological recovery with CD4 lymphocyte count <50 cells/mm^3^ at 6 months was associated with a significant increased risk of death (OR 1.8) and prescription of combination antiretroviral therapy was associated with increased survival (OR 0.2). In our study, patients that survived to the PJP episode and were adherent to HAART presented good long-term prognosis (90% survival at 5-years follow-up) compared to those patients with bad adherence to HAART (34% survival at 5 years follow-up, *P* < 0.001). IVDU as risk factor for HIV infection was the only factor associated with bad adherence during follow-up. The high rates of long-term survival in patients that recover from an episode of PJP are in contrast to those reported for patients with central nervous system opportunistic infections. In these patients, the 5-year survival has been reported to be poorer probably in relation to severe neurological sequelae.^31^

There are some limitations in our study. It is possible that some PJP cases from our demographical area may have not been detected if they received attention in other medical centers. Moreover, a 5-year follow-up was only achieved in 61 of the 121 patients included in the long-term survival analysis (Figure 2A). Anyway, our series represent the longer survival data in patients with PJP published to date.

In conclusion, despite PJP incidence has experienced a significant reduction in the HAART era, in-hospital mortality seems to remain stable. The proportion of patients with unknown HIV-infection at the time of PJP diagnosis and the mean age of the patients seem to be increasing in the last years, as a reflex of late HIV-infection diagnosis. Medical efforts must be directed to early diagnosis of HIV infection.
